# Proximal femoral fractures in children: incidence, complications, and functional outcomes—a population-based study from Finland

**DOI:** 10.2340/17453674.2025.44752

**Published:** 2025-09-30

**Authors:** Sini-Tuuli KOIVISTO, Ilkka HELENIUS, Antti STENROOS, Juho-Antti AHOLA, Topi LAAKSONEN

**Affiliations:** 1 Department of Pediatric Orthopaedics and Traumatology, University of Helsinki and Helsinki New Children’s Hospital, Helsinki; 2 Faculty of Medicine, University of Helsinki, Helsinki; 3 Department of Orthopaedics and Traumatology, University of Helsinki and Helsinki University Hospital, Helsinki; 4 Finnish Pediatric Orthopaedics Research Group (FiPO), Helsinki, Finland

## Abstract

**Background and purpose:**

Pediatric proximal femoral fractures are rare and frequently complicated fractures with avascular necrosis (AVN), nonunion, deformity, leg-length discrepancy (LLD), and premature physeal closure (PPC). Our aim was to describe the incidence, complications and functional outcomes.

**Methods:**

In this register-based study from a 10-year period (2014–2023) we identified 51 non-pathological proximal femoral fractures from the KIDS Fracture Tool database. Statistical yearbooks of Helsinki were utilized to estimate annual incidence. We used interviews and Oxford Hip Scores (OHS) for functional outcome assessment. If any symptom or functional deficit was described, or if Oxford Hip Score (OHS) was < 41, patients were also invited for clinical examination and radiography.

**Results:**

51 patients with a proximal femoral fracture (31 boys) were identified representing 0.2% (51/21,121) of all child fractures with a population-based annual incidence of 1.7/100,000 children. We interviewed 46/51 of the patients or their guardians via telephone. 6/46 were invited for clinical examination and radiography. Median follow-up of contacted patients was 4 (range 1–9.5) years. Complications occurred in 9/20 patients with collum and trochanteric fractures (pain from osteosynthesis 4, AVN 3, nonunion 1, coxa vara 1, LLD 1, PPC 0) and in 7/31 with subtrochanteric fractures (pain from osteosynthesis 5, misplaced pins 2, angular deformity 1, peri-implant fracture 1). All underwent reoperation. The median OHS was 48 (interquartile range 47–48) at last follow-up. Functional outcomes were impaired in 3 patients. All 3 had AVN.

**Conclusion:**

The incidence of non-pathological pediatric proximal femoral fractures is low. Despite frequent complications, impaired functional outcomes concerned only patients with AVN at median 4-year follow-up.

Proximal femoral fractures in pediatric populations are rare, and comprise < 1% of all fractures [1]. A population-based incidence of 0.5–4/100,000 children has been reported in a binational study including pathological and non-pathological fractures [2]. In contrast to femoral shaft fractures, proximal fractures occur more often in older children [3]. Increasing age predicts both higher incidence and higher involvement of boys [2]. These injuries often follow high-energy traumas (motor vehicle collisions, falls, sports), while low-energy fractures are associated with predisposing conditions or lesions, such as bone cysts [1].

Management of collum, trochanteric, and subtrochanteric fractures emphasizes reduction and fixation [4,5]; nevertheless, these fractures are associated with a high risk of complications [6]. This introduces the risk of long-term morbidity, and understanding these injuries’ significance to the patient in the longer term is important. Few studies have focused on reporting outcomes: Ratliff’s criteria [7] have been used for reporting outcomes for collum and trochanteric fractures, and outcomes for subtrochanteric fractures with Flynn’s criteria have been reported even less [8,9]. Most previous outcome reports are based on retrospective data and rely more on radiographic findings [6,8-11]. The aim of our study is to describe the incidence and complications, and to assess functional outcomes, by contacting those treated for pediatric proximal femoral fracture in a population-based study.

## Methods

### Study design

This is a descriptive, register-based follow-up study investigating population-based characteristics and outcomes of pediatric proximal femoral fractures. Eligible proximal femoral fractures from a 10-year-study period (January 1, 2014–December 31, 2023) were identified using the KIDS Fracture Tool. Reporting adhered to the STROBE guidelines [12].

### Setting

This study included pediatric proximal femoral fracture patients treated at the New Children’s Hospital during 2014–2023, where the management of these fractures is centralized. Our patients are from the Province of Uusimaa, which includes Helsinki capital region (Helsinki, Espoo, Vantaa, Kauniainen) and smaller municipalities. It is the only tertiary-level hospital in Helsinki capital region and the sole provider of on-call pediatric orthopedic treatment in Finland. The hospital services are accessible to all in our public-funded healthcare system.

### Data collection

The study population was identified using the KIDS Fracture Tool, which is an electronic database designed for pediatric fracture management and quality monitoring. This tool has prospectively recorded data (age, home municipality, injury mechanism, diagnosis, management, complications, follow-up visits) on fractures diagnosed in patients aged 15 years or younger since 2014 [13]. The total number of all children’s fractures during the study period was obtained from the KIDS Fracture Tool. Yearly counts of the pediatric population (< 16 years) between 2014 and 2023 in Helsinki capital region (Helsinki, Espoo, Vantaa, Kauniainen) were obtained from the Statistical Yearbook of Helsinki for annual incidence estimation [14]. Details from radiographs (e.g., fracture location, dislocation, angulation) and clinical contacts (e.g., status, recovery process) were recorded from patient records. Complications (e.g., AVN, nonunion, PPC, LLD, angular deformities) were re-evaluated from follow-up radiographs and/or computed tomography scans, if available. Outcome data was collected from patient interviews via telephone and, if needed, clinical examination and radiography.

### Study population

**Figure 1:**
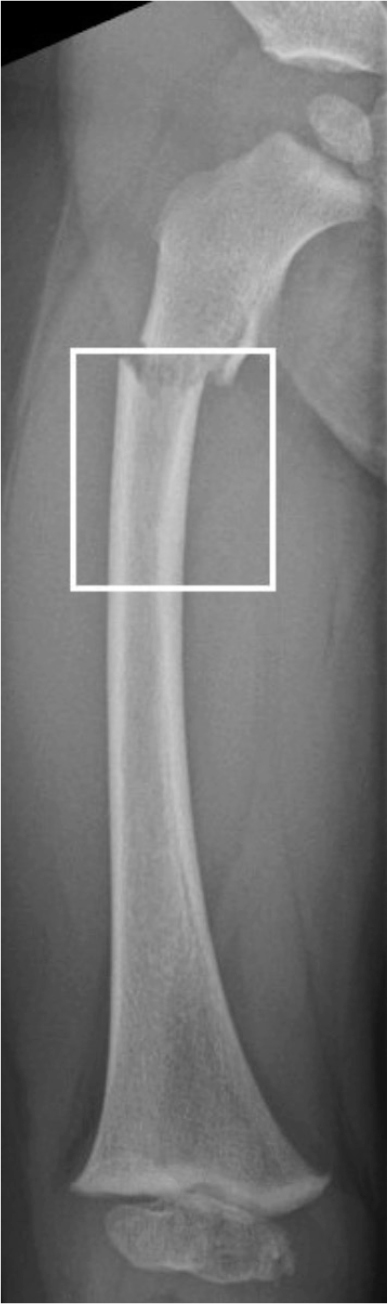
The area located within 20% of the total length of the femur below the lesser trochanter, defines the subtrochanteric fracture area. Subtrochanteric fractures by our definition should be entirely confined to this area, not only extend to it.

We reviewed radiographs of all proximal femoral fractures and diaphyseal fractures registered in the KIDS Fracture Tool to confirm diagnosis and eligibility. Patients with pathological fractures, slipped capital femoral epiphysis, stress fractures, or avulsion fractures were excluded. Collum and trochanteric fractures were classified using the Delbet classification, which includes transepiphyseal, transcervical, basicervical, and intertrochanteric fractures denoted by Delbet types I–IV, respectively [15]. Subtrochanteric fractures were defined as those occurring entirely within 20% of the total femoral length below the lesser trochanter (Figure 1). Subtrochanteric fractures were classified into complete and incomplete (torus, greenstick) fractures. Patients were followed for a minimum of 1 year and no patient was excluded due to this criterion.

### Variables

Injury mechanism was categorized into various falls (ground level and from a height), motor vehicle accidents (MVA), sports accidents, and others. Injury mechanisms were categorized into 3 levels of energy: (i) low energy (ground-level falls), (ii) moderate energy (MVAs at speeds below 30 km/hour or falls from heights under 3 meters), and (iii) high energy (MVAs exceeding 30 km/hour or falls from substantial heights). Dislocation was classified as non-displaced (≤ 2 mm) or displaced (> 2 mm) in either plane. AVN was defined as post-traumatic presentation of sclerosis, subchondral lucency, and femoral head collapse, and then further graded to Ratliff’s AVN categories I–III [7]. Nonunion was defined as presence of an unhealed fracture at 6 months [16]. PPC was defined as the presence of a physeal bar or bony bridge on CT in the absence of physiological physeal closure. Coxa vara and varus/valgus malunion in the subtrochanteric region were defined as 10° difference compared with the healthy side. LLD was defined as ≥ 2 cm difference between limbs [17].

### Outcomes

We attempted to contact all patients, or their guardians, if patients were < 18 years, to assess outcomes via telephone. Hip function was evaluated using the Oxford Hip Score (OHS) questionnaire (certified Finnish translation) [18]. The OHS assesses arthritis pain and functional deficit, ranging from 0 (worst outcome) to 48 (best outcome). Additionally, patients were invited to provide subjective descriptions of any pain, functional deficit, or dissatisfaction with the cosmetic outcome of their treatment. Those with OHS ≤ 41 or those reporting significant pain-related, functional, or cosmetic issues were invited for clinical follow-up and radiography. OHS > 41 was considered as a good result, as this represents the most conservative value found in the literature to denote the best outcome category [19].

### Statistics

Counts, percentages, and median with range or interquartile range (IQR) were used as descriptive statistics. Pairwise deletion was used for missing values. The 10-year cumulative annual incidence rates for all proximal femoral fractures, collum and trochanteric fractures, and subtrochanteric fractures were calculated for Helsinki capital area (Helsinki, Espoo, Vantaa, Kauniainen). Statistics were compiled using Microsoft Excel (Microsoft Corp, Redmond, WA, USA).

### Ethics, use of AI, funding, and disclosures

The study protocol was approved by the Helsinki University Hospital Review Board (HUS/564/2024). The KIDS Fracture Tool is linked to our electronic medical records, and written consent was not needed from parents for this register-based inquiry. Verbal informed consent was requested during interview. AI was not utilized. Author STK received a grant from the Finnish Medical Foundation to enable full-time working and declares no conflicts of interest. The other authors have not received any funding and declare no conflicts of interest. Complete disclosure of interest forms according to ICMJE are available on the article page, doi: 10.2340/17453674.2025.44752

## Results

**Figure 2:**
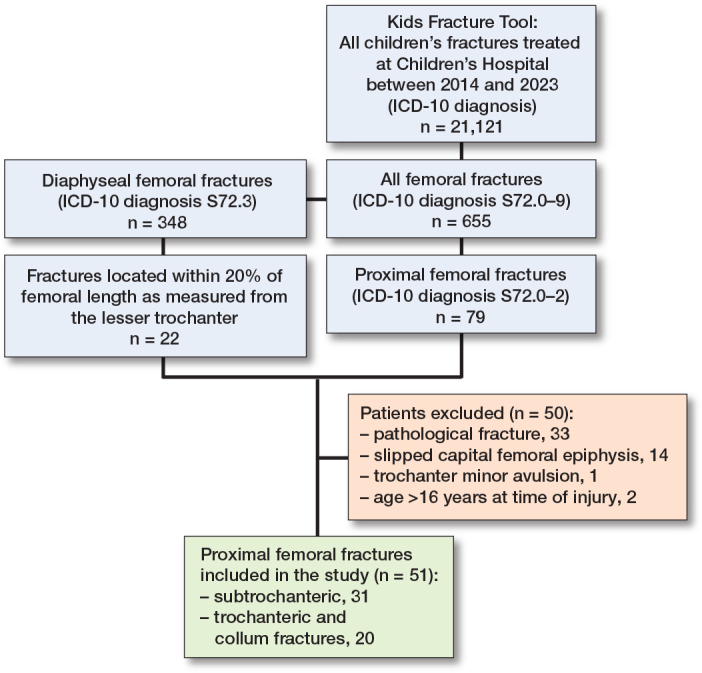
Patient flowchart.

During the 10-year-study period we found 79 proximal femoral factures and 348 diaphyseal femoral fractures from the KIDS Fracture Tool in the Province of Uusimaa, including Helsinki (Figure 2). Of these, 51 proximal femoral fractures (31 boys) met the inclusion criteria for the study (Figure 2). They comprised 18 collum, 2 trochanteric, and 31 subtrochanteric femoral fractures. The median age was 10.1 years (IQR 3.7–14.3). Proximal femoral fractures represented 0.2% (51/21,121) of all children’s fractures in the Province of Uusimaa. The population-based annual incidence was 1.7/100,000 in Helsinki capital area. The median follow-up period of all 51 patients was 3.6 years (range 1–9.5), and the median follow-up period of the 46 contacted patients was 4 years (range 1.1–9.5).

### Collum and trochanteric fractures

20 children (11 boys) presented with collum (n = 18) and trochanteric (n = 2) fractures. The fracture distribution according to Delbet classification from I–IV was 1, 11, 6, and 2, respectively. The median age was 13.5 years (IQR 10.5–15.3). The population-based annual incidence was 0.6/100,000 children. The most frequent injury mechanisms were 9 traffic accidents and 5 falls from a height (Table 1). 14 patients’ fractures were displaced: 1 with Delbet I, 7 with Delbet II, 5 with Delbet III, and 1 with Delbet IV fractures. In 2 patients, a non-dislocated and a 3 mm dislocated fracture were first unnoticed from plain radiographs, delaying diagnosis. 2 non-dislocated fractures were managed with cast immobilization in 1.3- and 8.7-year-old patients. 18 were managed with open reduction and internal fixation (ORIF) (Table 2).

**Table 1:** Characteristics of pediatric proximal femoral fractures

Mechanism of injury												
Factor	n	Median age (range)	Various falls		Traffic	Sport	Other	Injury energy			Associated injuries ^**a**^	Treated < 24 hours
			ground	height				Low	Moderate	High		
Collum and trochanteric fractures												
Delbet I	1	13.5	1					1				1
Delbet II	11	12.4 (8.7–15.5)	1	2	5	3		2	6	3	3	8
Delbet III	6	14.9 (12.0–15.6)		3	3				2	4	4	4
Delbet IV	2	8.2 (1.3–15.1)	1		1			1		1	1	2
Total	20	13.5 (1.3–15.6)	3	5	9	3		4	8	8	8	15
Subtrochanteric fractures												
Complete	25	9.5 (1.9–15.1)	4	10	5	6		6	16	3	1	22
Torus	5	1.8 (1–2.8)		2			3	5				4
Greenstick	1	1.6	1					1				1
Total	31	7.6 (1–15.1)	5	12	5	6	3	12	16	3	1	27
Total	51	10.1 (1–15.6)	8	17	14	9	3	16	24	11	9	42

a 4 patients suffered multiple associated fractures and internal organ injuries, 4 suffered from 1 other fracture, and 1 sustained laceration wound only.

**Table 2:** Characteristics of pediatric proximal femoral fractures by treatment method

Factor	n	Median age (range)	Median dislocation, mm (range) ^**a**^				
Management of collum and trochanteric fractures				Delbet I	Delbet II	Delbet III	Delbet IV
Cast in situ	2	5 (1.3–7.8)	1 (0–2)		1		1
PHP	2	10.6 (8.8–12.4)	23.5 (16–31)		1	1	
DHS	7	14.7 (12.1–15.5)	15 (0–35)		4	3	
Cannulated screws	8	13.9 (8.9–15.6)	10 (0–26)	1	5	2	
LFN	1	15.1	50				1
Total	20	13.5 (1.3–15.6)	14.5 (0–50)	1	11	6	2
Management of subtrochanteric fractures				Complete subtrochanteric		Torus	Greenstick
Limited weight-bearing	2	1.9 (1–2.8)	0			2	
Cast in situ	2	1.3 (1–1.6)	0			1	1
MUA and cast in situ	5	1.9 (1.8–3.7)	4 (0–18)	3		2	
Plate (LCP = 6, PHP = 5 **^**c**^**)	11	9.3 (2.8–12.9)	37 (7–49)	11			
FIN	7	9.5 (7.1–11)	25 (8–45)	7			
Locking nail (ALFN = 3, LFN = 1)	4	14.9 (14.5–15.1)	43 (12–100)	4			
Total	31	7.6 (1–15.1)	9 (0–100)	25		5	1

a 4 patients did not have lateral plane radiographs to evaluate sagittal displacement.

b 6 patients did not have lateral plane radiographs to evaluate sagittal displacement, and 1 patient did not have AP-plane radiographs to evaluate coronal displacement.

c One 2.8-year-old had preceding 7-day traction before PHP fixation.

ALFN: adolescent lateral femoral nail; DHS: dynamic hip screw; FIN: flexible intramedullary nails; LCP: locking compression plate; LFN: lateral femoral nail; MUA: manipulation under anesthesia; PHP: pediatric hip plate.

Complications occurred in 9/20 patients during follow-up (Table 3). All complications occurred to patients with Delbet II fracture. 8/9 of these patients underwent at least 1 unplanned reoperation, and in 1/9 patient reoperation is yet to come. Complications included 4 removals of osteosynthesis material due to pain, 3 AVNs, 1 nonunion, 1 coxa vara (from AVN), 1 hardware failure, and 1 LLD (from AVN), and 0 PPCs (Table 3). All AVNs were noticed 12 to 14 weeks post-injury.

**Table 3:** Clinical details of all patients with complications

Number	Sex, age	Fracture type	Mechanism of injury and associated injuries	Dislocation (mm)	Treatment, fixation, and timing	Complication	Reoperation	OHS	Total follow-up (years)
1	Girl, 12.4	Delbet II	MVA, distal femur fracture	15	DHS < 24 hours	Ratliff I—AVN, loss of alignment, coxa vara, LLD	Re-fixation with cannulated screws and removal of screws	47	5.4
2	Girl, 10.1	Delbet II	Horse-riding accident	23	Cannulated screws < 24 hours	Ratliff II—AVN	Underwent THA	14	1.4
3	Girl, 8.7	Delbet II	Horse-riding accident, clavicle fracture	≤ 2 **^**b**^**	Cast in situ, cannulated screws 12 days post-injury due to loss of reduction	Ratliff II—AVN	Not yet Further management strategy of AVN is still under consideration	42	1.3
4	Boy, 14.3	Delbet II	Ski-jumping accident	20	Cannulated screws < 12 hours	Non-union	DHS fixation due to non-union	48	9.1
5	Boy, 8.9	Delbet II	Soccer	26	Cannulated screws < 24 hours	Hardware failure	Replacement with DHS fixation	48	1
6	Girl, 8.8	Delbet II	Ski-jumping accident	31	PHP < 24 hours	Pain	Unplanned OR	NA **^**a**^**	1.9
7	Boy, 15.5	Delbet II	Moped accident	≤ 2 **^**b**^**	DHS < 24 hours	Pain	Unplanned OR	48	4.8
8	Boy, 15.5	Delbet II	Bike accident	22	Screw fixation < 24 hours	Pain	Unplanned OR	42	2.6
9	Boy, 13.4	Delbet II	Bike accident, wrist fracture	≤ 2 **^**b**^**	Initially missed diagnosis, DHS 30 days later	Missed diagnosis, pain	Unplanned OR	48	7.2
10	Boy, 10.5	CS	Swing accident	47	LCP	Multiple peri-implant fractures	Multiple redo surgeries	NA **^**a**^**	3.1
11	Girl, 9.5	CS	Quad bike accident	28	FIN	Suboptimal pin placement, varus deformity, and pain	Redo surgery x2, unplanned OR	48	9.1
12	Boy, 7.6	CS	Sledding accident	45	FIN	Suboptimal pin placement	Redo surgery	48	5
13	Girl 7.2	CS	Downhill skiing accident	8	FIN	Pain	Unplanned OR	45	4.3
14	Boy, 9.8	CS	Sledding accident	44	FIN	Pain	Unplanned OR	48	2.9
15	Boy, 10.9	CS	Downhill skiing	25	FIN	Pain	Unplanned OR	48	7.8
16	Boy, 9.7	CS	MVA	32	LCP	Pain, whilst excessive scar tissue resection	Unplanned OR	48	1.7

a Not available due to inability to reach patient.

b Non-dislocated

AVN: avascular necrosis of the femoral head as classified by Ratliff into types I–III; CS: Complete subtrochanteric DHS: dynamic hip screw; FIN: flexible intramedullary nail; LCP: locking compression plate; LLD: leg-length discrepancy; MVA: motor vehicle accident; OR: osteosynthesis removal; PHP: pediatric hip plate; THA: total hip arthroplasty.

### Subtrochanteric fractures

31 children (20 boys) with a median age of 7.6 years (IQR 2.8–10.9) presented with subtrochanteric fractures. The population-based annual incidence was 1.1/100,000 children. The most frequent injury mechanisms were 12 falls from a height and 5 sports accidents traffic (see Table 1). All complete fractures (25/31) were displaced. 9 subtrochanteric fractures were treated by cast immobilization. 21 complete fractures underwent ORIF and 1 closed reduction and internal fixation (CRIF) (see Table 2).

Complications occurred in 7/31 patients (see Table 3). All complications occurred to patients with complete subtrochanteric fractures, and they underwent at least 1 unplanned reoperation. Complications included 5 removals of osteosynthesis due to pain, 1 varus malunion, 2 suboptimal pin placements, and 1 sustaining multiple peri-implant fractures.

### Outcomes

We contacted 46/51 (90%) patients: 18 with collum and trochanteric and 28 with subtrochanteric fractures. Their median OHS was 48 (IQR 47–48), and outcome impairments were found in 3 patients, all with AVN: patients 1, 2, and 3 (Tables 3 and 4). 7 patients were invited for clinical examination and radiography due to subjective concern in the affected hip/limb (7/7) and due to OHS ≤ 41 (1/7). These subjective concerns were interpreted as relevant to preceding trauma or its management in 2 patients with AVN (nos 2 and 3) and in 1 patient with prominent osteosynthesis (no. 7). Patient no. 2 scored an OHS of only 14 (Table 4).

**Table 4:** Details and clinical findings of the 7 patients invited for clinical examination and additional radiograph

Number	Sex, age	Fracture type	Injury mechanism	Treatment and complications	OHS	Follow-up (years)
2	Girl, 10.1	Delbet II	Horse-riding accident	Cannulated screws fixation within 24 hours. Developed AVN	14	1.4
	Patient’s concern: Stiffness, abnormal gait, inability to bear weight due to pain.			
	Clinical findings: Abnormal gait, ROM **^**a**^**: hip flexion 20°, internal and external rotations absent. Hip extension 0°. In extended hip, internal rotation 10°, external rotation 30°.			
	Radiography: AVN, femoral head partially collapsed.			
	Conclusion: Severely disabling AVN. Soon after our clinical examination the patient underwent THA.			
3	Girl, 8.7	Delbet II	Horse-riding accident	Cast in situ, cannulated screw 12 days post-injury due to loss of reduction. Developed AVN	42	1.3
	Patient’s concern: Abnormal gait which is accentuated at distances > 200 m. No rest pain or need for painkillers.			
	Clinical findings: Abnormal gait. ROM: hip flexion 130°, internal rotation 40°, external rotation 30°, hip extension 0°. In extended hip, external rotation 0°.			
	Radiography: AVN, femoral head partially collapsed.			
	Conclusion: Absence of hip extension contributes to the abnormal gait. Will require further surgery. Management strategy of AVN still under consideration.			
7	Boy, 15.5	Delbet II	Moped accident	DHS, now wants plate removal	48	4.8
	Patient’s concern: Post-exertional abnormal sensation distal to trochanter major.			
	Clinical findings: Normal hip function. Slim patient, DHS fixation is palpable.			
	Radiography: No findings.			
	Conclusion: Excellent hip recovery, osteosynthesis likely bothers, will proceed with removal.			
8	Boy, 15.5	Delbet II	Bike accident	Cannulated screws removed due to pain	42	2.6
	Patient’s concern: Pain along gluteal region, especially after immobilization.			
	Clinical findings: Normal hip function			
	Radiography: No findings.			
	Conclusion: Excellent hip recovery. Able to participate in various sports. Symptoms seem unrelated to hip trauma.			
17	Girl, 15.3	Delbet III	MVA, multiple fractures	DHS	45	3.5
	Patient’s concern: Occasional hip pain and stiffness.			
	Clinical findings: Normal hip function.			
	Radiography: Prominent lesser trochanter.			
	Conclusion: Excellent hip recovery. Currently at renovation-related work the hip does not bother or cause limitations Symptoms seem unrelated to hip trauma.			
11	Girl, 9.5	CS	Quad bike accident	FIN, suboptimal pin placement, osteosynthesis removal due to pain	48	9.1
	Patient’s concern: Affected hip and leg seem weaker at sports activities.			
	Clinical findings: 10–15° internal rotation deficit in affected hip.			
	Radiography: 11° varus malunion.			
	Conclusion: Excellent hip recovery. Reported weakness seems unrelated to hip trauma.			
13	Girl, 7.2	CS	Downhill skiing	FIN, osteosynthesis removal due to pain	45	4.3
	Patient’s concern: Pain in ipsilateral knee and heel.			
	Clinical findings: Normal hip function Affected leg 10 mm longer. Palpation tenderness in the heel.			
	Radiography: No findings.			
	Conclusion: Excellent hip recovery. Limb discrepancy clinically insignificant. Symptoms seem nunrelated to hip trauma: clinical examination suggests apophysitis in knee and heel.			

a Range of motion (passive)

For abbreviations, see Table 3.

**Figure 3:**
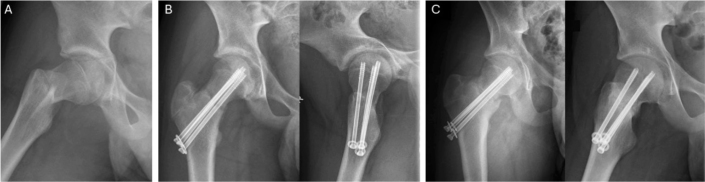
(A) Primary radiograph showing a dislocated Delbet II fracture, (B) fracture alignment following operation, and (C) non-union 7 months post-injury

39 patients were interviewed only, without follow-up invitation: 79% (31/39) scored 48 on OHS, and the remaining 21% (8/39) scored ≥ 44. Among them, patient no. 1 with Ratliff I AVN scored 47 on OHS at 5-year follow-up, despite the fact that on last clinical examination 3 years post-accident, gait was found to be affected, with significant range of motion (ROM) limitations (flexion 80°, internal rotation 5°, external rotation 40°, and abduction 10°) and total collapse of the femoral head. However, during the interview she expressed having hip pain 1–2 times a month with no limitations in daily activities due to impaired hip function. Regardless of this discrepancy between objective findings and subjective view on good outcome, we considered the patient’s outcome as impaired due to her limited ROM, abnormal gait, and radiographic deformity. Patient no. 4 with nonunion scored 48 on OHS at 9-year follow-up and described no symptom/functional deficit (Figure 3). Patient no. 5 with hardware failure following screw fixation of Delbet II fracture scored 48 on OHS at 1-year-follow up. 4 patients with uncomplicated recovery described minor issues during interview: 2 had asymmetric gait (complete subtrochanteric), 1 had mild pain during long-distance running (complete subtrochanteric), and 1 had hip stiffness (Delbet II). 3 patients were dissatisfied with the cosmetic outcome of their treatment because of excessive scar formation.

## Discussion

Prior annual incidence of non-pathological proximal femoral fractures has not been reported previously. Our aim was to describe the population-based incidence, complications, and functional outcomes after pediatric proximal femoral fractures in a register-based follow-up study. We found 51 proximal femoral fractures accounting for 2/1,000 pediatric fractures during the study period, with a population-based annual incidence of 1.7/100,000 children. Complications occurred in 16 children, all requiring reoperation. Functional outcomes were satisfactory in 43/46 patients contacted, with impairments found only in patients with AVN.

In contrast to our study, an older Finnish study reported a somewhat higher incidence of 0.6–1/100,000 in < 7-year-old children and 1.5–4/100,00 in 7–16-year-old children, which can be attributed to inclusion of pathological fractures. A Turkish study found a lower incidence of 0.45/100,000 in < 16-year-old children despite inclusion of pathological fractures [20], which may reflect a difference in predisposing factors in different populations. High-energy injury mechanisms such as falls from a height, MVAs, and sports injuries prevailed, which is in line with previous reports [21,22].

**Figure 4:**
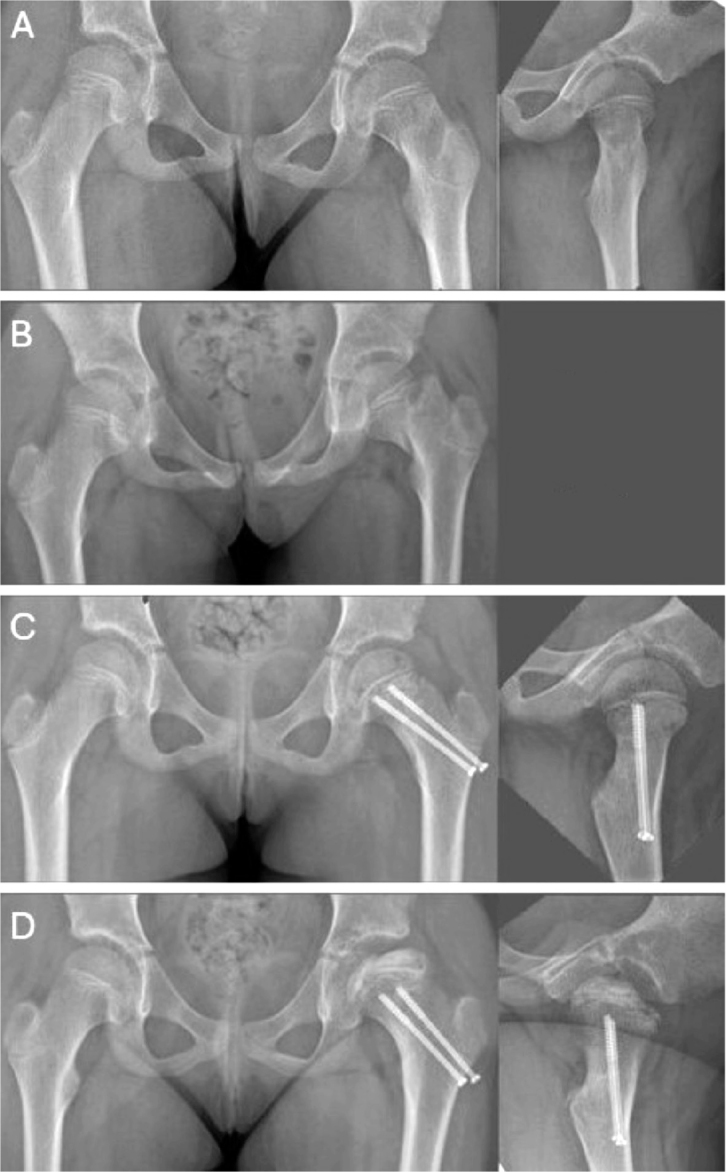
(A) Primary radiographs showing a non-dislocated Delbet II fracture in an 8-year-old girl. (B) Loss of alignment at 10 days’ follow-up. (C) Ratliff type 1 AVN at 3 months post-injury. (D) AVN at 11 months post-injury.

Pediatric collum and trochanteric fractures are currently approached in good time, with an emphasis on anatomic reduction and internal fixation, by open reduction, if necessary, to mitigate severe complications such as AVN, coxa vara, and nonunion [4,16,22]. However, a uniform management strategy is yet to be defined. In our study, we encountered a failure of initial nonoperative management of a collum facture, leading to AVN (Figure 4). For us, this emphasizes the importance of internal fixation even in non-displaced femoral collum fractures.

Collum and trochanteric fractures are still complicated by AVN (23%), nonunion (11%), coxa vara (18%), PPC (22%), and LLD (11%) [6]. Our incidences for AVN (15%), non-union (5%), coxa vara (5%), LLD (5%), and PPC (0%) were somewhat lower than previously reported [6]. In addition to small sample size and aggressive operative approach, differences can reflect heterogeneity of complication definitions across studies [6]. While AVN and nonunion are well defined in the pediatric literature [7,16], definitions for coxa vara and LLD were viewed as controversial in our study. We thought complications should be considered as events causing short- or longer-term harm to the child. Coxa vara of > 10°compared with the unaffected side has demonstrated significant difference in Harris Hip Score in 1 study [10], so this definition was chosen. LLD has been defined as a discrepancy of > 1 cm in some studies. We defined LLD as a discrepancy of ≥ 2 cm. In our experience the gait remains unaffected with LLD between 1 and 2 cm, and in the longer term, LLD > 2 cm is often reported as problematic, while the significance of discrepancies below 2 cm is not well established [17].

Definition of complete subtrochanteric fractures of the femur has been versatile in the literature. The latest definition by Pombo and Shilt defined a fracture as subtrochanteric if the fracture line extends to an area that is within 10% of the length of the femur when measured from the lesser trochanter [11]. However, for this study, our patients had subtrochanteric fracture if the fracture line was located entirely within 20% of femoral length as measured from the lesser trochanter, as we view these fractures as different in their fixation nature, with inserting muscles pulling the proximal fragment in flexion, abduction, and external rotation, as opposed to the long oblique fractures of the diaphysis.

The rotational forces of the hip muscles pose a challenge in maintaining closed anatomic reduction of dislocated subtrochanteric fractures, so reduction and fixation is currently opted for [5,8,11]. Our approach reflected this. Complications of complete subtrochanteric fractures include angular deformity (especially varus malunion), nail irritation, and LLD [9]. There is no definition for significant varus/valgus malunion in pediatric subtrochanteric fractures. We used a > 10° definition for varus malunion, a definition used in pediatric femoral shaft fractures, though this is based on general agreement rather than evidence of long-term harm [23]. Flexible intramedullary nails (FIN) have been concluded to be a viable option for school-aged children, but inferior to plating regarding angular deformities [5,9]. Our only varus malunion followed FIN fixation. We noted that the more proximally the subtrochanteric fracture was located, the more likely it was that the surgeon opted for plate fixation, reflecting concerns over FIN stability.

Functional outcomes of collum and trochanteric fractures have mostly been evaluated with criteria set by Ratliff, evaluating pain, movement, activity, and radiographic findings [7]. A meta-analysis including studies between 1960 and 2010 reported good outcomes in 60%, fair in 19%, and poor in 21% of patients, with proximity of the fracture in Delbet classification predicting worse outcomes [6]. Within AVN patients, Ratliff reported worst outcomes with type I AVN (total collapse of femoral head and neck) and more favorable in types II–III (partial collapse) [7]. Outcomes of subtrochanteric fractures have mostly been evaluated with titanium elastic nails (TEN) outcome scoring, which evaluates LLD, malalignment, pain, and complications [8]. These outcomes have mostly been assessed in the context of assessing FIN use or comparing FIN and plate fixation, and reported outcomes have typically been excellent in > 50% of patients [8,9,11].

We used OHS, interviews, and, if needed, clinical examination for outcome assessment in both fracture groups, with an attempt to better reflect patients’ perspective of outcomes, as previously used Ratliff’s and Flynn’s criteria have placed more emphasis on radiographic findings [7,8]. Our OHSs, interview, and clinical examination findings were predominantly very good. We found impaired outcomes only in 3 patients with AVN. This results in a good functional outcome of 83% (15/18) in contacted collum and trochanteric patients, which may reflect our comparably smaller incidence of major complications [6]. Good outcomes applied to 100% (28/28) of contacted subtrochanteric patients.

### Limitations

First, a few children might have been treated entirely in the private sector, which may affect the incidence. However, pediatric proximal femoral fractures are almost invariably treated at our tertiary-level university hospital. Second, although we interviewed 46/51 children, we did not invite all to our clinic for an outcome assessment. However, the invitation threshold was low. Our OHSs may reflect a ceiling effect, which is common even among validated PROMs [24]. Acknowledging that OHS is not validated in children or parents as proxies, we included interviews, where patients or parents could express any functional deficit or symptom (that the OHS would not, perhaps, be able to measure). There is no validated pediatric hip PROM in available in Finnish. An English version exists, without recommendation for clinical use [25]. Our good outcomes in 93% (43/46) of contacted patients should be interpreted with caution, as only a subset of patients were invited for clinical examination and radiography, and these conditions carry risk for longer-term morbidity that cannot be assessed in the current study. Coxa vara and LLD have been linked to osteoarthritis [17,26], and long-term effects of varus malunion > 10° are unknown. Finally, injury energy classification was based on subjective judgement.

### Conclusion

The incidence of non-pathological pediatric proximal femoral fractures is low. Despite frequent complications, impaired functional outcomes only concerned patients with AVN at a median of 4 years’ follow-up.

## Notes

STK: study design, data acquisition, statistics, manuscript preparation, correspondence. IH: study design, manuscript preparation. AS: study design, data acquisition, manuscript preparation. JAA: data acquisition, manuscript preparation. TL: study design, data acquisition, manuscript preparation,

Handling co-editors: Cecilia Rogmark and Robin Christensen

*Acta* thanks Ole Rahbek and other anonymous reviewers for help with peer review of this manuscript.
